# The transcriptional analysis of pepper shed light on a proviral role of light-harvesting chlorophyll a/b binding protein 13 during infection of pepper mild mottle virus

**DOI:** 10.3389/fpls.2025.1533151

**Published:** 2025-01-27

**Authors:** Weihong Lin, Shugen Zhang, Hao Zhang, Xiaomei Deng, Tong Jiang, Xifeng Chen, Laihua Dong, Qin Yan, Lianyi Zang, Yongping Xing, Zhenquan Wang, Qin Zhang, Kaitong Du, Huolin Shen, Junmin Zhang, Tao Zhou

**Affiliations:** ^1^ Key Laboratory for Pest Monitoring and Green Management of Ministry of Agriculture and Rural Affairs, and Department of Plant Pathology, China Agricultural University, Beijing, China; ^2^ Laboratory of Plant Tissue Culture Technology of Haidian District, Beijing, China; ^3^ College of Agriculture and Biology, Liaocheng University, Liaocheng, China; ^4^ College of Horticulture, China Agricultural University, Beijing, China

**Keywords:** differentially expressed gene, transcriptomic analysis, chlorophyll ab binding protein 13, virus-induced gene silencing, photosynthesis, flavonoid biosynthesis

## Abstract

Pepper mild mottle virus (PMMoV), a member of the genus *Tobamovirus*, causes severe damage on pepper worldwide. Despite its impact, the pathogenicity mechanisms of PMMoV and the pepper plant’s response to infection remain poorly understood. Here, we compared the transcriptomic changes in a susceptible pepper inbred line 21C241 with a resistant inbred line 21C385 seedlings, following systemic PMMoV infection using RNA sequencing. Our results revealed that PMMoV induced more pronounced mosaic symptoms and higher viral accumulation levels in the susceptible line 21C241 compared to the resistant line 21C385. We identified 462 and 401 differentially expressed genes (DEGs) in the systemically-infected leaves of the susceptible and resistant lines, respectively, when compared to their healthy counterparts. The majority of these DEGs were involved in photosynthesis and the biosynthesis of secondary metabolites, with 28 DEGs exhibiting distinct expression patterns between the two lines. Notably, the expression level of the *chlorophyll a-b binding protein 13* (*CAB13*) was significantly up-regulated in resistant line 21C385 following PMMoV infection. Functional analysis through silencing of *CAB13* in pepper and *Nicotiana benthamiana* demonstrated a reduction in PMMoV accumulation, suggesting that CAB13 plays a positive role in facilitating PMMoV infection in pepper plants. Taken together, our findings highlight the distinct gene expression profiles between susceptible and resistant pepper lines in response to PMMoV infection and confirm the proviral role of CAB13. This study provides valuable insights into the molecular mechanisms underlying resistance and susceptibility in pepper plants and may inform future strategies for disease management.

## 1 Introduction

Pepper (*Capsicum annuum* L.) plants, renowned for their important economic value as a solanaceous vegetable crop, are susceptible to a variety of pathogens, including viruses, fungi, and bacteria (Kim et al., 2018; Spiegelman and Dinesh-Kumar, 2023). Viral infections could particularly cause serious pepper yield and quality reductions under both field and protective cultivation conditions (Peng et al., 2015). Over 45 distinct viral species have been documented to infect pepper crops (Kenyon et al., 2014). The pepper mild mottle virus (PMMoV) stands out as a particularly severe threat to pepper production, attributed to its high contagiousness and long persistence in soil (Kenyon et al., 2014). On susceptible inbreds, PMMoV infection initially causes mild foliar mosaic symptoms followed by mottling and malformation of leaves and fruits, resulting in considerable losses to both yield and economic value (Kim et al., 2012). Reports indicate that over half of the pepper cultivation areas in Northeast China were affected by PMMoV, with the epidemic covering up to 15 ha and resulting in a 30% yield loss in 2014 (Kim et al., 2018).

PMMoV, belonging to genus *Tobamovirus* of the family *Virgaviridae*, possesses a positive-sense single-stranded RNA (+ssRNA) genome (Adams et al., 2009; Lefkowitz et al., 2018). The genome of PMMoV consists of 6356–6357 nucleotides (nts), containing a m^7^GpppG cap structure at 5′ end and a 3′ terminal structure that mimics tRNA. It encodes four proteins: p126, which functions as a viral suppressor of RNA silencing; p183, an RNA-dependent RNA polymerase; a movement protein; and a coat protein (CP), all of which play crucial roles in the virus’s life cycle (Adams et al., 2009). Most recently, molecular studies have uncovered that PMMoV infection induces autophagy in host cells, and the CP counters the antiviral defense by interfering with the self-interaction of the chloroplast outer membrane protein 24 (OMP24) (Qin et al., 2014; Han et al., 2023). Despite these findings, comprehensive understanding of the pathogenicity mechanisms of PMMoV remains limited.

Considering the intricate nature of host-virus interactions that encompass a myriad of physiological processes, transcriptomic analysis has emerged as an essential tool for elucidating the underlying mechanisms (Lu et al., 2012). RNA sequencing (RNA-Seq) is a powerful technique for identifying genes whose expression profiles are modulated in response to both biotic and abiotic stresses. For example, RNA-Seq has been used to study global gene expression profiles and signal transduction pathways in plant defense responses to various stresses, including virus infection (Miozzi et al., 2014; Tian et al., 2016). A recent comparative transcriptome study in wheat (*Triticum aestivum*) infecting wheat dwarf virus has shed light on the impact of viral infection on phytohormone signaling and photosynthetic metabolic pathways (Liu et al., 2020). Integrated single-molecule long-read sequencing and Illumina sequencing revealed the resistance mechanism of *Psathyrostachys huashanica* in response to barley yellow dwarf virus-GAV (Shen et al., 2020). With the complete sequencing of the pepper genome (Qin et al., 2014), we are now equipped to investigate transcriptomic alterations in pepper plants in response to PMMoV infection.

Virus-induced gene silencing (VIGS) is a useful tool for pepper functional genomics. To date, several grass-infecting RNA viruses and DNA viruses have been modified and used as VIGS vectors for pepper (Ascencio-Ibanez and Settlage, 2007; del Rosario Abraham-Juarez et al., 2008; Oh et al., 2010). Among these reported VIGS vectors, the tobacco rattle virus (TRV)-based VIGS vector has been successfully applied to functionally characterize pepper genes responding to bacterium and virus infections (Kim et al., 2006; Yang et al., 2022; Han et al., 2023). Here, we chose the TRV-based VIGS vector to rapidly screen for proteins with functions in the interaction between pepper plants and PMMoV.

In this study, we conducted an RNA-Seq analysis to examine the transcriptomic changes in both a susceptible pepper inbred line 21C241 and a resistant line 21C385 plants following PMMoV infection. We identified differentially expressed genes (DEGs) that are significantly enriched in pathways of photosynthesis and biosynthesis of secondary metabolites. Furthermore, our study highlights the proviral role of light-harvesting chlorophyll a/b binding protein 13 (CAB13) in the context of PMMoV infection.

## 2 Materials and methods

### 2.1 Plant growth and virus inoculation

Seeds of resistant inbred line 21C385, susceptible line 21C241 pepper and *N. benthamiana* seeds were grown inside a growth chamber set at 25°C and a 16 and 8 h (light/dark) photoperiod and 60% humidity. PMMoV-16.9 isolate was from a previously preserved source in our lab. For virus inoculation, approximately 0.2 g of PMMoV-infected leaf tissues were harvested and homogenized in 0.01 M phosphate buffer (pH 7.0) at 1:2 (w/v) ratio and centrifuged at 4500 g for 5 min at 4°C. The supernatants were mechanically inoculated onto the upper two young leaves of four-leaf stage pepper plants. At 13 dpi, the upper inoculated young leaves were harvested, frozen in liquid nitrogen, and then stored at -80°C till further use. Leaves of plants inoculated with phosphate buffer only were also harvested at 13 dpi and used as the non-infected controls.

### 2.2 RNA-seq

In this study, we conducted three independent virus inoculation experiments, pooling the upper young leaves from at least three individual pepper plants for each of the PMMoV-infected and non-infected groups. RNA-Seq libraries were prepared using 1μg total RNA per reaction and a TruSeq RNA sample preparation kit. Double-stranded cDNAs were synthesized using the SuperScript double-stranded cDNA synthesis kit (Invitrogen) and random hexamer primers (Illumina). After quantification using a TBS380 mini-fluorometer, the paired-end RNA-Seq libraries were sequenced on an Illumina HiSeq Xten platform (2×150 bp read length). The resulting clean reads were aligned to the *Capsicum annuum* cv CM334 reference genome according to their orientation using the TopHat software (http://tophat.cbcb.umd.edu/, version2.0.0). To identify DEGs between the two treatment groups, the expression level of each transcript was calculated based on the number of fragments per kilobase of exons per million mapped reads (FRKM). RSEM (http://deweylab.biostat.wisc.edu/rsem/) was used to determine gene abundance. The R statistical package software edgeR (empirical analysis of digital gene expression in R, http://www.bioconductor.org/packages/2.12/bioc/html/edgeR.html) was used for differential expression analysis. Functional enrichment analyses, including GO and KEGG, were performed to identify the enriched DEGs in the GO terms and metabolic pathways at Bonferroni-corrected *p* < 0.05, compared to the whole-transcriptome background. GO functional enrichment and KEGG pathway analyses were performed using Goatools (https://github.com/tanghaibao/Goatools) and KOBAS (http://kobas.cbi.pku.edu.cn/home.do), respectively.

### 2.3 Plasmid construction

For VIGS assays, a partial fragment of *NbCAB13* (200 bp) and *CaCAB13* (200 bp) was amplified and inserted individually into pTRV2 vector. Briefly, these fragments were amplified individually from an *N. benthamiana* and *C. annuum* cDNA through PCR using specific primers (**Supplementary Table 1**). The resulting PCR fragments were digested with the *Xba I* and *Kpn I* restriction enzymes and cloned individually into the pTRV2 plasmid to produce pTRV2:*NbCAB13* and pTRV2:*CaCAB13*, respectively. Sequences of *NbCAB13* (Niben101Scf02971Ctg037) and *CaCAB13* (CA07g18220), were retrieved from the GenBank database (https://solgenomics.net/).

### 2.4 Total RNA extraction and gene expression analysis by RT-qPCR

To validate the RNA-Seq results, 6 DEGs were selected and tested for their expressions through RT-qPCR. We extracted RNA from both infected and healthy pepper samples using the TransZol reagent (TransGen, Beijing, China), followed by RNase-free DNase I treatment (Tiangen, Beijing, China). Concentrations and qualities of the isolated RNA samples were monitored using a NanoDrop2000 spectrophotometer (Thermo Fisher Scientific, Waltham, USA). Synthesis of cDNA was done using 2 μg total RNA per sample, an oligo(dT) primer, random primers and a gRNA remover in a 20 μL reaction (Aidlab, Beijing, China). RT-qPCR was performed using the Universal SYBR qPCR Master Mix (Vazyme) with the ABI 7500 Real Time PCR system (Applied Biosystems). The PCR reactions were subjected to an initial denaturation step of 94°C for 30 s, followed by 40 cycles of 95°C for 5 s and 60°C for 34 s. Primers used in qPCR amplifications were designed according to the gene sequences from the *Capsicum annuum* cv CM334 (http://peppersequence.genomics.cn) and RNA-Seq data. The expression levels of *C. annuum Ubiquitin-conjugating protein* gene *(CaUBI-3*, AY486137) or *N. benthamiana Actin* gene (*NbActin*, AY179605) was used as internal controls, respectively. Relative expression levels of these genes were calculated using the 2^-ΔΔCT^ method. Differences between the treatments were analyzed using Student’s *t* test. All the experiments were conducted at least three times.

### 2.5 Virus-induced gene silencing in *C. annuum* and *N. benthamiana*

To silence *NbCAB13*, pTRV2:*NbCAB13* was co-expressed with pTRV1 in *N. benthamiana* plants by agroinfiltration. pTRV2:mCherry combined with pTRV1 served as the control. The cultures were infiltrated individually into leaves of 3-4 leaf stage *N. benthamiana* plants. Plants silenced for 6 days were inoculated with PMMoV-GFP. For silencing of *CaCAB13* in pepper, pTRV2:*CaCAB13* and pTRV1 were mixed at a 1:1 ratio and agro-infiltrated onto 2-leaf stage pepper leaves. Pepper plants silenced for 18 days were inoculated with PMMoV.

### 2.6 Western blot analysis

Pepper soluble proteins were extracted with buffer (220 mM Tris-HCl, pH 7.4, 250 mM sucrose, 1 mM MgCl_2_, 50 mM KCl) containing β-mercaptoethanol (10 mM). The protein concentration was measured by protein assay kit (Tiangen, Beijing, China). The yielded extracts were loaded and separated by 10% sodium dodecylsulphate-polyacrylamide gel electrophoresis (SDS-PAGE), and electroblotted onto nitrocellulose membranes; immunodetection was performed using antibodies specific for PMMoV CP and *β-actin* (EASYBIO, Beijing, China), and detected by chemiluminescence using an eECL Western Blot Kit (CWBIO, Jiangsu, China) according to the manufacturer’s protocol. The relative expression levels of individual proteins on the immunoblots were quantified using the ImageJ image analysis tool (http://imagej.net/).

## 3 Results

### 3.1 PMMoV-infection differences in pepper susceptible inbred line 21C241 and resistant line 21C385 plants

**Figure 1:**
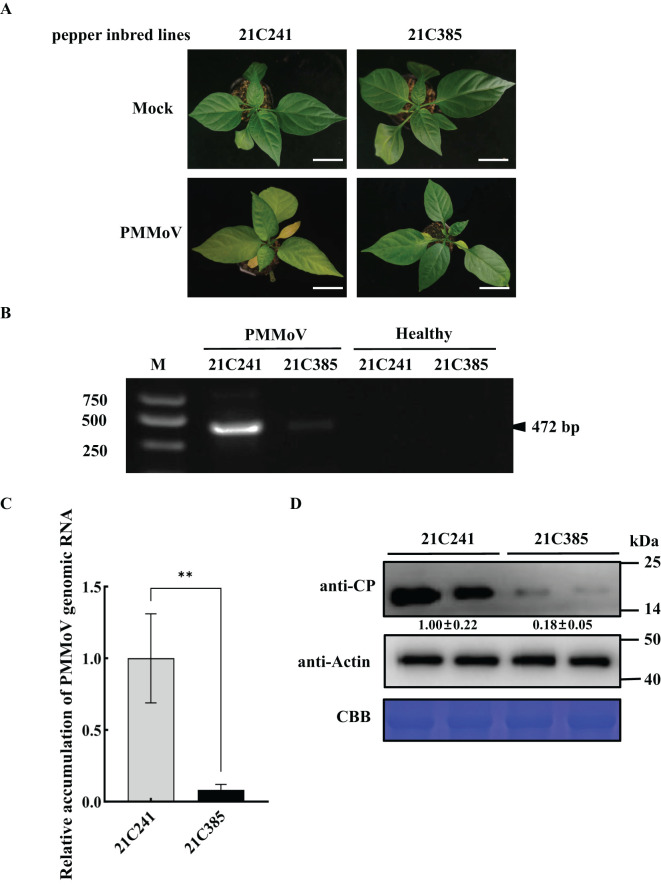
PMMoV infection on the seedlings of susceptible pepper inbred line 21C241 and resistant line 21C385. **(A)** Typical symptoms on pepper seedlings. The plants were photographed at 13 days post inoculation (dpi). Mock, inoculated with phosphate buffer. Bar = 5 cm. **(B)** Detection of PMMoV in 21C241 or 21C385 seedlings by reverse transcription-polymerase chain reaction (RT-PCR). (M, DL2000 Marker). **(C)** RT–quantitative PCR (RT-qPCR) of the PMMoV genomic RNA. Asterisks indicated statistically different (**P ≤ 0.01), determined by the two-tailed Student’s *t* test, error bars were standard error of the mean (SEM). These experiments were performed with three independent biological replicates (n = 3) with similar results. **(D)** Western blotting showing the accumulation levels of PMMoV coat protein (CP). Accumulations of pepper actin in these tissues were used as loading controls. CBB, Coomassie brilliant blue.

The susceptible pepper inbred line 21C241 and resistant line 21C385 seedlings were mechanically inoculated with an isolate of PMMoV with 98.65% genomic identity to isolate PMMoV-16.9. The inoculated seedlings were periodically monitored for disease symptom development. By 13 days post inoculation (dpi), systemically infected leaves of susceptible line 21C241 showed clearly foliar yellowing and distortion, while resistant line 21C385 showed no obvious symptoms (**Figure 1A**). Semiquantitative reverse transcription PCR (RT-PCR) assays indicated that PMMoV was detected with relatively high level in susceptible 21C241 pepper, while with low level in resistant 21C385 plants, and was not detected in mock-inoculated plants (**Figure 1B**). Reverse transcription–quantitative PCR (RT-qPCR) and immunoblotting results verified that the accumulation levels of PMMoV genomic RNA and CP were significantly higher in the upper leaf of susceptible 21C241 than that of resistant 21C385 (**Figures 1C, D**).

### 3.2 Sequencing and *de novo* assembly of transcriptome

To study the gene expression alternations of the two inbred lines following PMMoV infection, the upper systemically-infected leaves of mock-inoculated and PMMoV-inoculated susceptible line 21C241 and resistant 21C385 were separately harvested at 13 dpi. Total RNA was isolated, and mRNA were prepared. Totally, 12 libraries were constructed from healthy and PMMoV-infected pepper leaf samples with three replicates for each sample, and then were subjected to RNA-Seq.

A total of 82.23 GB raw reads were obtained from these libraries (i.e., 43,287,672 to 43,916,148, 45,008,026 to 48,946,638, 40,462,338 to 52,341,680 and 46,203,088 to 50,896,532 raw data for PMMoV-infected and healthy groups, respectively) (**Table 1**). After removing the low-quality reads and adapter sequences, the clean reads ranged from 43,279,658 to 43,908,206 for PMMoV-infected susceptible 21C241 group, and 44,998,550 to 48,937,076 for PMMoV-infected resistant 21C385 group, and 40,454,306 to 52,333,118 for healthy susceptible 21C241 group, and 46,193,146 to 50,884,142 for healthy resistant 21C385 group. Among these reads, the percentage of reads mapped to the pepper reference genome (CM334) was over 79.89%, 84.40%, 80.38%, and 84.36% for PMMoV-infected susceptible 21C241, resistant 21C385, and their healthy groups, respectively. For each library, more than 90.09% of the clean reads had a quality score at Q30 (sequencing error rate less than 0.1%), and the GC content of the obtained reads ranged from 45.24 to 47.43% (**Table 1**).

**Table 1:** Summary of RNA-Seq data.

Sample	Raw reads	Clean reads	Total mapped	Multiple mapped	Unique mapped	Q30 (%)	GC Contents (%)
Healthy 21C385-1	50,102,190	50,089,610	42,366,051 (84.58%)	6,728,081 (13.43%)	35,637,970 (71.15%)	95.87	47.12
Healthy 21C385-2	50,896,532	50,884,142	42,923,900 (84.36%)	7,622,276 (14.98%)	35,301,624 (69.38%)	95.91	47.43
Healthy 21C385-3	46,203,088	46,193,146	39,047,091 (84.53%)	4,434,596 (9.60%)	34,612,495 (74.93%)	91.79	45.45
PMMoV-infected 21C385-1	48,946,638	48,937,076	41,568,897 (84.94%)	6,088,908 (12.44%)	35,479,989 (72.50%)	90.79	45.91
PMMoV-infected 21C385-2	45,584,770	45,577,198	38,659,883 (84.82%)	5,475,611 (12.01%)	33,184,272 (72.81%)	90.09	45.45
PMMoV-infected 21C385-3	45,008,026	44,998,550	37,980,101 (84.40%)	6,228,796 (13.84%)	31,751,305 (70.56%)	91.23	46.46
Healthy 21C241-1	44,738,182	44,728,708	35,954,915 (80.38%)	4,547,247 (10.17%)	31,407,668 (70.22%)	91.46	46.05
Healthy 21C241-2	52,341,680	52,333,118	43,236,295 (82.62%)	5,316,661 (10.16%)	37,919,634 (72.46%)	93.77	46.18
Healthy 21C241-3	40,462,338	40,454,306	33,136,596 (81.91%)	4,538,591 (11.22%)	28,598,005 (70.69%)	90.19	46.02
PMMoV-infected 21C241-1	43,515,484	43,506,890	35,571,114 (81.76%)	2,917,156 (6.71%)	32,653,958 (75.05%)	90.78	45.24
PMMoV-infected 21C241-2	43916,148	3,908,206	35,080,171 (79.89%)	3,445,418 (7.85%)	31,634,753 (72.05%)	91.84	45.92
PMMoV-infected 21C241-3	43,287,672	43279658	34,586,333 (79.91%)	4,869,423 (11.25%)	29,716,910 (68.66%)	90.77	47.08

### 3.3 Identification of DEGs in susceptible and resistant pepper plants following PMMoV infection

**Figure 2:**
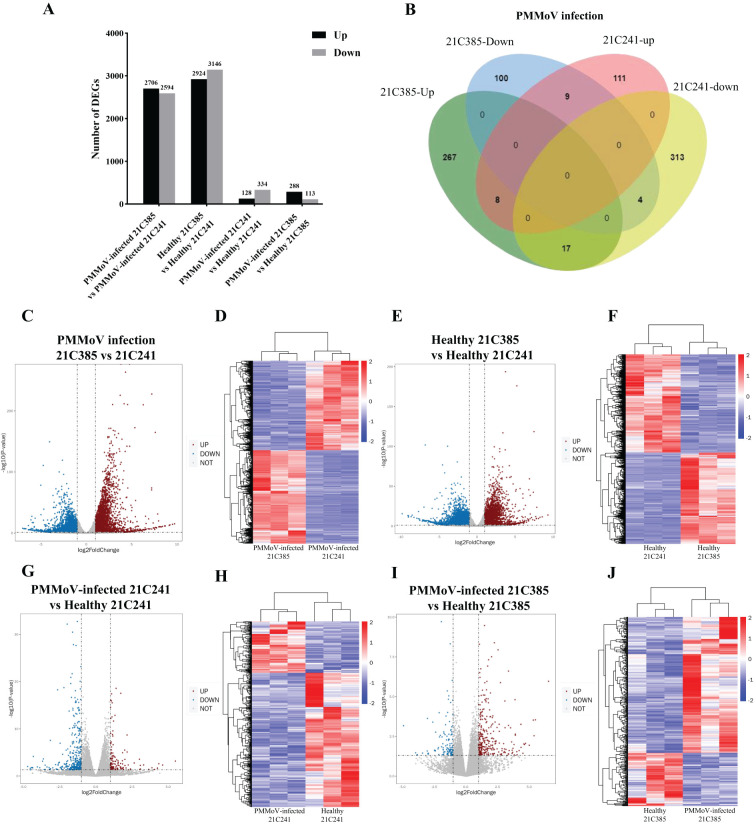
Genes differentially expressed between PMMoV-infected and healthy 21C241 and 21C385 pepper plants. **(A)** Total numbers of up-regulated and down-regulated gene. **(B)** Venn diagram of up- and downregulated genes in pepper plants following PMMoV infection. **(C, E, G, I)** Volcano plots showing differentially expressed genes (DEGs) in four groups. The red and blue colors represent the significantly up- and down-regulated genes, respectively (*P <*0.05 and |log_2_ (FC)| ≥ 1). **(D, F, H, J)** Hierarchical clustering of DEGs based on the log_10_ FPKM values. The color scale (blue to red) represents gene expression intensities (log_10_ FPKM, low to high).

To identify genes that might play important roles during PMMoV infection in pepper plants, we analyzed the transcriptomic profiles of these 12 libraries and presented the expression level of each gene as FPKM (expected number of Fragments Per Kilobase of transcript sequence per Millions base pairs sequenced). DEGs were identified by comparing gene expression between PMMoV-inoculated and mock-inoculated samples using the criteria (*P <*0.05 and |log_2_ (FC)| ≥ 1). Totally, there were 7,481 DEGs between the healthy and PMMoV-infected peppers in the two inbred lines. A total of 5,300 DEGs were identified in resistant 21C385 *vs* susceptible 21C241 group following PMMoV infection, among which 2,706 (51.06%) were up-regulated, and 2594 (48.94%) were down-regulated (**Figures 2A, C**). The transcriptomic profiles and hierarchical clustering of DEGs in PMMoV-infected resistant 21C385 *vs* susceptible 21C241 is illustrated in **Figure 2D**. At the same time, a total of 6070 DEGs were identified between healthy resistant and susceptible plants, of which 2924 (48.17%) showed up-regulated and 3146 (51.83%) down-regulated (**Figures 2A, E, F**). Moreover, 462 DEGs were identified in susceptible 21C241 following PMMoV infection, which included 128 (27.71%) up-regulated and 334 (72.29%) down-regulated genes (**Figures 2A, G, H**). At the same time, comparative transcriptome analysis of resistant 21C385 following PMMoV infection generated 401 DEGs, and of all DEGs, 288 (71.82%) were significantly up-regulated, while 113 (28.18%) were down-regulated (**Figures 2A, I, J**). There were 38 same DEGs in the two lines responding to PMMoV infection, among which 26 displayed different and 12 displayed consistent responses (**Figure 2B**). Interestingly, eight up-regulated DEGs and four down-regulated DEGs were common to both lines (**Figure 2B**). Overall, susceptible line 21C241 showed more gene alternations than resistant line 21C385 following PMMoV infection.

### 3.4 Functional enrichment analysis of DEGs

Gene Ontology (GO) assignment was performed to classify the gene function of DEGs. Both up-regulated and down-regulated DEGs can be categorized into three main categories, including biological process (BP), cellular component (CC) and molecular function (MF). GO enrichment analysis was carried out using a threshold value (*P* ≤ 0.05). Three main categories of the GO classification, ‘photosynthesis’, ‘thylakoid’ and ‘electron transporter’ were the significantly enriched terms in comparing resistant line to susceptible line following PMMoV infection (**Supplementary Figure 1**). On healthy plants, GO enrichment results for DEGs between resistant and susceptible samples indicated that photosynthesis was the most significantly represented groups in the biological process category. Consistently, photosystem was the most commonly enriched terms in the cellular component (**Supplementary Figure 2**). On susceptible plants, the top three GO terms enriched were iron ion binding, transition metal ion binding and photosynthesis, light reaction (**Supplementary Figure 3**), whereas the top three GO terms enriched in resistant plants were photosynthesis, intracellular organelle part and organelle part (**Supplementary Figure 4**).

Further, analysis with Kyoto Encyclopedia of Genes and Genomes (KEGG) database indicated that some of the highly ranked pathways, including biosynthesis of secondary metabolites, photosynthesis, porphyrin and chlorophyll metabolism and metabolic pathways were significantly enriched in resistant line comparing to susceptible line in healthy plants or PMMoV-infected plants (**Supplementary Figures 5**, **6**). In susceptible plants, the three most significantly enriched pathways were GABAergic synapse, Axon guidance and Hippo signaling following PMMoV infection (**Supplementary Figure 7**). In resistant plants, metabolic pathways were the most enriched pathway items following PMMoV infection (**Supplementary Figure 8**).

### 3.5 Expression of genes involved in photosynthesis and chlorophyll metabolism

**Figure 3:**
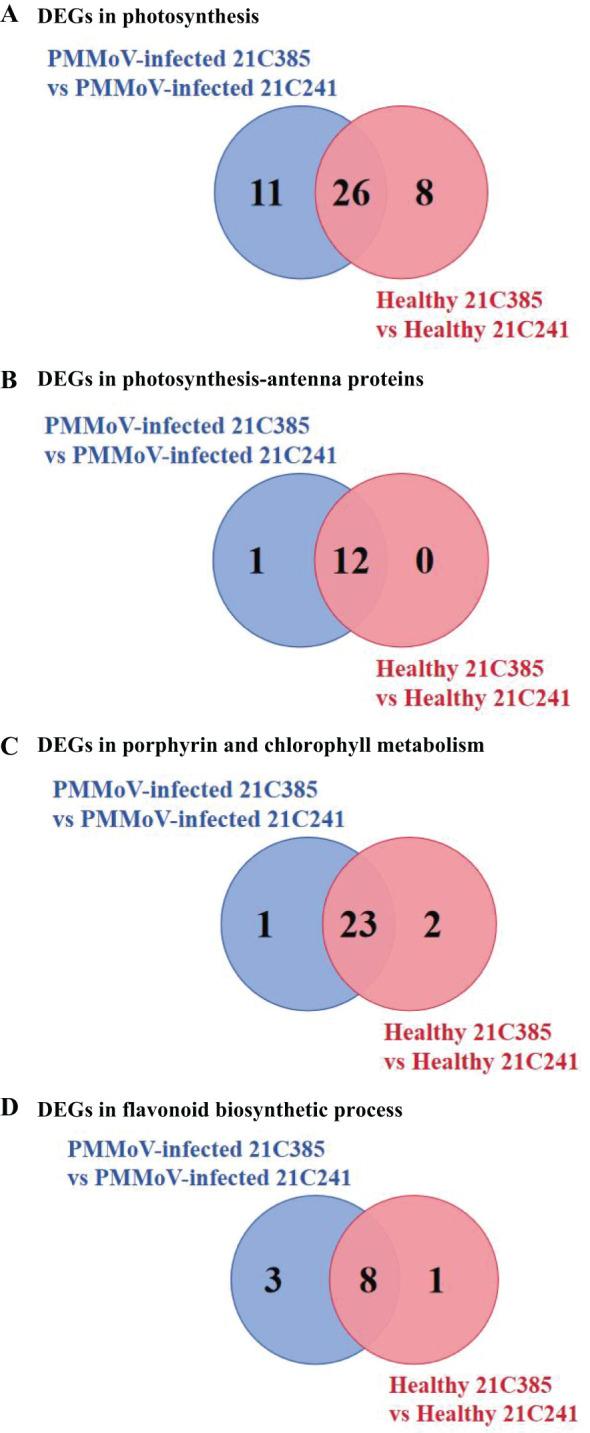
Specific DEGs involved in photosynthesis **(A)**, photosynthesis-antenna proteins **(B)**, porphyrin and chlorophyll metabolism **(C)** and flavonoid biosynthetic process **(D)** in PMMoV-infected and healthy 21C241 and 21C385 pepper plants.

Previous studies revealed that virus infection can modify photosynthesis, and disturb chloroplast components and functions (Kyselakova et al., 2011; Manfre et al., 2011; Krenz et al., 2012). To elucidate the changes in the chloroplast following PMMoV infection, we analyzed the expression pattern of the DEGs related to photosynthesis and chloroplast between susceptible and resistant lines. As shown in **Supplementary Figures 1**, **2**, the related DEGs were mainly involved in categories corresponding to thylakoid (GO:0009579), photosystem II (GO:0009523), chloroplast thylakoid (GO:0009534) and photosynthetic membrane (GO:0034357). The main genes involved in photosynthesis, photosynthesis-antenna proteins and porphyrin and chlorophyll metabolism are listed in **Table 2**. Comparing to susceptible plants, there were 37 and 34 DEGs involved in photosynthesis in resistant plants following PMMoV-infection and healthy, respectively, among which 26 were up-regulated consistently (**Figure 3A**). Comparing to susceptible plants, PMMoV-infection caused up-regulation of 11 genes, encoding cytochrome b6/f complex subunit IV (CA01g14310), Photosystem I chlorophyll a apoprotein A2 (CA01g29040), ATP synthase CF1 alpha subunit (CA03g01980), photosystem I P700 apoprotein A2 (CA03g02160), ferredoxin (CA05g19620) and Photosystem II reaction center W protein (CA09g01280). In healthy resistant plants, comparing to susceptible plants, eight genes, encoding photosystem II protein L (CA09g10720), photosynthetic NDH subunit of lumenal location (CA10g00150, CA10g08630), oxygen-evolving enhancer protein (CA10g15660), Photosystem I chlorophyll a apoprotein A1 (CA11g11050), ATP synthase delta chain (CA12g12950) and photosystem I P700 apoprotein A1 (CA12g14160), were up-regulated. Notably, 12 DEGs in the photosynthesis-antenna protein pathway were significantly up-regulated, all of which encode chlorophyll a-b binding proteins and are related to photosynthesis. One (CA04g23570), encoding chlorophyll a-b binding protein of LHCII type 1, was up-regulated in PMMoV-infected resistant plants compared to susceptible plants (**Figure 3B**). In addition, the porphyrin and chlorophyll metabolism pathways were represented, which contain many genes involved in chlorophyll biosynthesis, such as Geranylgeranyl diphosphate reductase (CA03g29990), oxygen-dependent coproporphyrinogen-III oxidase (CA10g06010) and magnesium-chelatase subunit ChlI (CA10g03710) were consistently and greatly increased. CA10g11860 was up-regulated in PMMoV-infected resistant plants compared to susceptible plants. Two genes (CA03g07320, CA07g18790), were down-regulated in healthy resistant plants compared to susceptible plants (**Figure 3C**).

**Table 2:** List of differentially expressed genes related to photosynthesis and chlorophyll metabolism in PMMoV-infected and healthy 21C385 and 21C241 pepper plants.

Pathway	Gene name	Description	Log_2_FC (21C385 *vs* 21C241)	
			PMMoV-infected	Healthy
photosynthesis	CA04g00010	Ferredoxin-1	1.7218	2.0409
	CA06g09290	photosystem I reaction center subunit II	2.6247	2.3664
	CA01g23080	ATP synthase subunit b	3.5886	2.3763
	CA06g26270	photosystem I reaction center subunit XI	2.1975	2.3753
	CA04g14360	photosystem I P700 chlorophyll a apoprotein A1	2.7236	2.2644
	CA04g21580	photosystem I reaction center subunit IV B	2.7018	2.7329
	CA01g25930	cytochrome f	3.2916	1.8415
	CA02g17690	oxygen-evolving enhancer protein 3-2	2.584	2.7236
	CA01g23070	Photosystem II CP43 reaction center protein	2.8032	1.892
	CA03g11060	ATP synthase gamma chain	1.616	1.5848
	CA08g04280	photosystem II CP43 chlorophyll apoprotein	2.7261	2.1519
	CA07g20940	photosystem I reaction center subunit V	2.61	2.2887
	CA07g21500	photosystem II 10 kDa polypeptide	2.3816	2.535
	CA06g28140	Photosystem I reaction center subunit IV	2.7817	2.4848
	CA01g20410	ATP synthase CF1 alpha subunit	2.3344	3.0657
photosynthesis- antenna protein pathway	CA01g17090	chlorophyll a-b binding protein CP26	2.6935	2.3404
	CA03g29950	chlorophyll a-b binding protein P4	2.5333	2.4718
	CA04g00760	chlorophyll a-b binding protein 3C	2.5296	2.6627
	CA07g18220	chlorophyll a-b binding protein 13	2.9	2.8499
porphyrin and chlorophyll metabolism pathway	CA05g05340	Glutamate-1-semialdehyde 2,1-aminomutase	1.5822	2.2962
	CA10g06010	oxygen-dependent coproporphyrinogen-III oxidase	1.6519	2.4055
	CA01g25500	divinyl chlorophyllide a 8-vinyl-reductase	1.8503	1.4233
	CA03g29990	Geranylgeranyl diphosphate reductase	1.9453	1.9277
	CA04g10380	Chlorophyllide a oxygenase	1.6295	1.3725

### 3.6 Expression of genes involved in biosynthesis of secondary metabolites

Plants synthesize a variety of secondary metabolites that function in plant protection (Zaynab et al., 2018), and our results above showed that several secondary metabolite pathways were significantly enriched (**Supplementary Figures 5**, **6**). Interestingly, flavonoid biosynthetic process (ko00941) KEGG pathway was enriched in both inbred lines, i.e. 9 DEGs were identified in healthy resistant plants comparing to susceptible plants, and 10 DEGs were in response to PMMoV infection, among which seven were down-regulated consistently (**Table 3**; **Figure 3D**). Three genes (CA03g30250, CA06g25930, CA10g22120) were down-regulated in PMMoV-infected resistant plants compared to susceptible plants. Two genes (CA02g14470 and CA12g19630) were down-regulated in healthy resistant plants compared to susceptible plants in **Table 3**. These results suggest that flavonoid metabolites might play an important part in resistance of pepper to PMMoV infection.

**Table 3:** List of DEGs related to photosynthesis and chlorophyll metabolism in PMMoV-infected and healthy 21C385 and 21C241 pepper plants.

Pathway	Gene name	Description	Log_2_FC (21C385 *vs* 21C241)	
			PMMoV-infected	Healthy
Flavonoid biosynthesis	CA02g14470	caffeoyl-CoA O-methyltransferase-like		-5.1397
	CA03g02050	photosystem I reaction center subunit II	-2.1726	-1.4015
	CA03g29350	flavonoid	-4.7392	-4.612
	CA05g17060	chalcone synthase 2	-1.8315	-1.0614
	CA09g02560	Flavonol synthase/flavanone 3-hydroxylase	-1.9729	-1.7481
	CA09g07820	acetyl-CoA-benzylalcohol acetyltransferase-like	-2.9837	-2.151
	CA11g02280	chalcone–flavonone isomerase isoform X1	-1.4192	-1.318
	CA11g14880	agmatine coumaroyltransferase-2-like	-1.4386	-1.6901
	CA12g19630	vinorine synthase-like		-4.4998
	CA03g30250	shikimate O-hydroxycinnamoyltransferase	-1.6558	
	CA06g25930	trans-cinnamate 4-monooxygenase	-1.0724	
	CA10g22120	Cytochrome 98A2	-1.9139	

### 3.7 Validation of transcriptomics data by RT-qPCR

**Figure 4:**
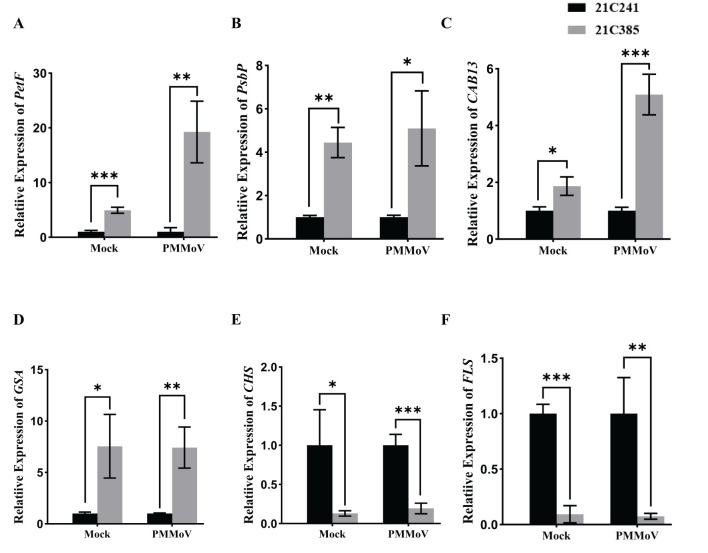
Validation of transcriptome results by RT-qPCR. Six genes, including *PetF*, *PsbP*, *CAB13*, *GSA*, *CHS* and *FLS*, were selected from the RNA-Seq data for RT-qPCR. Asterisks indicated statistically different (*P ≤ 0.1; **P ≤ 0.01; ***P ≤ 0.001), determined by the two-tailed Student’s *t* test, error bars were SEM. These experiments were performed with three independent biological replicates (n = 3) with similar results. Black represents 21C241 and, gray represents 21C385.

To validate the RNA-Seq results above, six DEGs were selected for RT-qPCR analyses using specific primers (**Supplementary Table 1**), including four up-regulated DEGs, [*PetF* and *PsbP* (photosynthesis), *CAB13* (photosynthesis-antenna protein), *GSA* (porphyrin and chlorophyll metabolism)], and two down-regulated genes, [*CHS* and *FLS* (flavonoid biosynthetic process)] in PMMoV-infected groups and healthy groups. In general, the RT-qPCR data of all selected genes were consistent with the DEG results from RNA-Seq (**Figure 4**), indicating that changes in gene expression determined by RNA-Seq were accurate.

### 3.8 Silencing of *CAB13* inhibits PMMoV infection in pepper and *Nicotiana benthamiana*

Genes in photosynthesis play diverse roles in virus infection (Cheng et al., 2008; Song et al., 2009). In higher plants, light capture of chlorophyll a/b binding proteins functions in the plant’s response to biotic and abiotic stresses (Liu et al., 2020). Our RNA-Seq data above showed that *CaCAB13* was up-regulated following PMMoV infection in resistant 21C385. To study the role of CAB in PMMoV infection, we used TRV-induced gene silencing (VIGS) in pepper and *N. benthamiana* (Liu et al., 2002).

**Figure 5:**
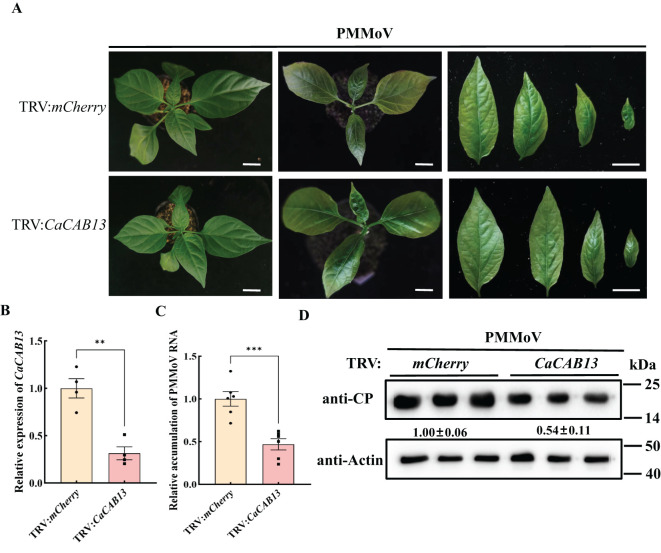
Silencing of *CaCAB13* inhibited PMMoV infection in pepper. **(A)** Milder yellowing symptoms on the upper leaves of TRV-induced gene silencing of *CaCAB13* compared with non-silencing control plants at 30 dpi of PMMoV. Bar = 5 cm. **(B)** RT-qPCR analysis of the silencing efficiency of *CaCAB13*. *CaUbi* was used as reference gene. Asterisks indicated statistically different (***P ≤ 0.001), determined by the two-tailed Student’s *t* test, error bars were SEM. These experiments were performed with three independent biological replicates (n = 3) with similar results. **(C)** Relative accumulation of PMMoV RNA in silenced and non-silenced plants at 30 days post PMMoV inoculation. Asterisks indicated statistically different (**P ≤ 0.01), determined by the two-tailed Student’s *t* test, error bars were SEM.These experiments were performed with three independent biological replicates (n = 3) with similar results. **(D)** Accumulation levels of PMMoV CP in upper leaves of TRV: *mCherry* and TRV: *CaCAB13* plants as detected by western blotting. Actin represented loading control.

A 200 bp fragment of *CaCAB13* was selected to specifically silence this gene, and was inserted into pTRV2 of TRV, producing TRV: *CaCAB13*. At 18 dpi of TRV: *CaCAB13* inoculation, the expression of *CaCAB13* decreased to 30% of the normal level in the control plants inoculated with TRV:*mCherry*, while the plants did not show any obvious changes (**Figures 5A, B**). Plants were then rub-inoculated with PMMoV. At 30 dpi of PMMoV infection, the *CaCAB13*-silenced plants had milder mosaic symptoms with less yellowing than the control plants (**Figure 5A**). We analyzed PMMoV RNA and CP accumulation levels at 30 dpi. Results showed that the accumulation level of PMMoV genomic RNA in the *CaCAB13*-silenced plants was significantly lower than that in control plants infected with the control vector, TRV:*mCherry* (**Figure 5C**). Immunoblotting results were consistent with the RT-qPCR results, and showed approximately 35% decrease in PMMoV CP accumulation in *CaCAB13*-silenced plants compared with that in control plants at 30 dpi (**Figure 5D**).

**Figure 6:**
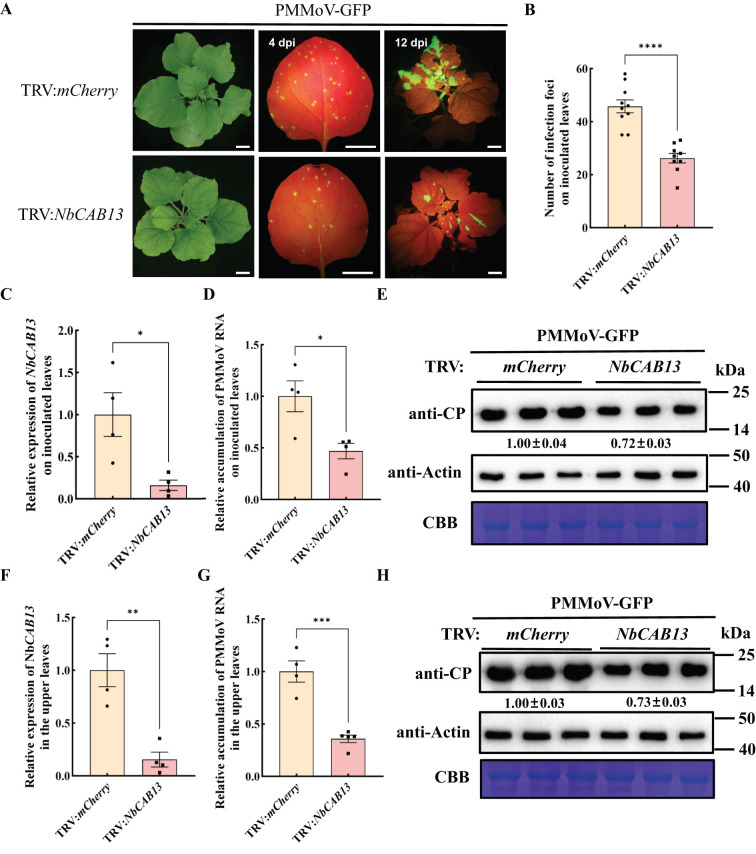
Silencing of *NbCAB13* inhibits PMMoV infection in *Nicotiana benthamiana*. **(A)** GFP fluorescence of PMMoV-GFP infection in the inoculated and upper leaves at 4 dpi and 12 dpi under UV light. Bars = 2 cm. **(B)** Number of infection foci on inoculated leaves at 4 dpi of PMMoV-GFP infection. Error bars show the mean ± SD of three replicates (at least 12 plants per replicate). The mean expression values were analyzed using Student’s *t* test (****P ≤ 0.0001). **(C**, **F)** RT-qPCR analysis of the silencing efficiency of *NbCAB13* in the inoculated **(C)** and systemic leaves **(F)**. *Nbactin* was used as reference gene. Asterisks indicated statistically different (*P ≤ 0.05, **P ≤ 0.01), determined by the two-tailed Student’s *t* test, error bars were SEM.These experiments were performed with three independent biological replicates (n = 3) with similar results. **(D**, **G)** Relative accumulation of PMMoV RNA in inoculated leaves **(D)** and systemically infected leaves **(G)** of TRV:*mCherry* and TRV: *NbCAB13* plants. Asterisks indicated statistically different (*P ≤ 0.05, ***P ≤ 0.001), determined by the two-tailed Student’s *t* test, error bars were SEM.These experiments were performed with three independent biological replicates (n = 3) with similar results. **(E, H)** Western blot showing the decreased accumulation of PMMoV CP in inoculated leaves **(E)** and systemically infected leaves **(H)** of TRV: *mCherry* and TRV: *NbCAB13* plants. Actin represented loading control. CBB, Coomassie brilliant blue.

We identified a homologue of *CaCAB13* in *N. benthamiana*, namely *NbCAB13*, with 92.73% nucleotide identity and 97.36% amino acid identity (**Supplementary Figures 9**, **10**). Next, we investigated the role of *NbCAB13* for PMMoV infection using the TRV-VIGS system in *N. benthamiana*. To specifically silence *NbCAB13*, a 200 bp fragment was cloned into pTRV2. Silencing of *NbCAB13* decreased the transcript level of *NbCAB13* to 16% while did not cause obviously developmental change in the plants (**Figures 6A, B**). Plants were then rub-inoculated with PMMoV-GFP (Han et al., 2023). Compared with TRV:*mCherry*-treated control plants, less fluorescent foci appeared on the inoculated leaves of *NbCAB13*-silenced plants at 4 dpi of PMMoV-GFP infection (**Figures 6A, C**). On the inoculated leaves, RT-qPCR results showed approximately 50% decrease of PMMoV genomic RNA level in *NbCAB13*-silenced plants compared with control plants. Immunoblotting results were consistent with the RT-qPCR results and showed approximately 40% decrease of PMMoV CP accumulation in *NbCAB13*-silenced plants compared with control plants (**Figures 6D, E**). Moreover, the speed of systemic infection was remarkably slower in *NbCAB13*-silenced plants than in the non-silenced control plants (**Figure 6A**). The expression level of *NbCAB13* decreased to 15% of the control plants in the upper leaves at 12 dpi of PMMoV (**Figure 6F**). RT-qPCR and immunoblotting showed that the accumulation levels of PMMoV genomic RNA and CP in the upper leaves of *NbCAB13*-silenced plants were significantly less than that in the non-silenced control plants (**Figures 6G, H**). Taken together, knockdown of *CAB13* gene expressions inhibited PMMoV accumulation in both pepper and *N. benthamiana*, suggesting the pro-viral role of CAB13 for PMMoV infection.

## 4 Discussion

Though PMMoV threatens pepper crops and causes economic losses worldwide (Kim et al., 2012), the information about host gene expression alternations in susceptible line and resistant line is scarce. In this study, we used RNA-Seq to compare gene expression changes in PMMoV-systemically infected leaves from a susceptible line and a resistant line. Following PMMoV infection, there were no infection symptoms in the upper leaves of resistant line 21C385, but the upper leaves of susceptible line 21C241 showed clear infection symptoms. Viral multiplication level was also lower in the upper leaves of 21C385 than in those of 21C241. These data suggest that the ability of PMMoV to mount a systemic infection and to move to the upper leaves was repressed in resistant line 21C385.

We identified 7,481 DEGs in total between the healthy and PMMoV-infected peppers in the two lines, and these genes may therefore have roles in resistance to PMMoV infection. Many of the DEGs were associated with photosynthesis and secondary metabolite biosynthesis. These results were consistent with a previous study in which PMMoV infection of susceptible peppers activated defense-response genes that could promote production of antimicrobial secondary metabolites to limit further spread of the virus (Jiao et al., 2020). Another study examined the transcriptomic responses of two pepper genotypes to PMMoV infection (Zhang et al., 2023). Two DEGs separately encoding dihydroflavonol 4-reductase (*DFR*) and chalcone synthase (*CHS*), which closely associate with the synthesis of flavonoid compounds, were identified. The expression of *CHS* was up-regulated in the tolerant genotype while down-regulated in susceptible genotype, suggesting to be responsible for the difference in disease resistance (Zhang et al., 2023). Nonetheless, our analysis showed that the expression of *CHS* was down-regulated in resistant line comparing with susceptible line following PMMoV infection or in healthy plants. We did not find the expression difference of DFR in all comparisons.

Both susceptible line 21C241 and resistant line 21C385 seedlings could be infected by PMMoV, but 21C241 showed obvious symptoms, whereas no significant symptoms were observed in 21C385. It is possible that both lines have the same pattern-recognition receptors that respond to virus infection, and that 21C385 shows asymptomatic because of the presence/activity of specific metabolic pathways that inhibit virus multiplication and transmission. From what has been mentioned above, GO enrichment analysis revealed enrichment of the flavonoid biosynthesis pathway in 21C385. Flavonoid compounds act as defenses against viruses (Seo et al., 2018), and many studies have shown that flavonoid accumulation is an important mechanism by which plants resist pathogen infection (Dong and Lin, 2021). For example, most anthocyanin biosynthetic genes, including *CHS*, chalcone isomerase (*CHI*), flavanone 3-hydroxylase (F3H), flavonoid 3’ -hydro-xylase (*F3’H*), *DFR*, anthocyanidine synthase (*ANS*) and UDP-glucose flavonoid 3-O-glucosyl transferase (*UFGT*) homologs, were highly up-regulated in ToCV-infected leaves, which displayed purple coloring symptoms (Seo et al., 2018). Two genes, (*DFR* and *CHS*), showed opposite responses to infection in resistant and susceptible pepper genotypes (Zhang et al., 2023). We also identified twelve DEGs that are closely associated with the synthesis of flavonoid compounds. Two DEGs, (*FLS* and *CHS*) the first committed enzyme in flavonoid biosynthesis, respectively, was down-regulated in the resistant 21C385. The difference in their expression between the two lines may be responsible for the difference in disease resistance. These results suggest that flavonoid compounds may play an important part in the resistance of 21C385 to PMMoV infection. Nonetheless, how flavonoids could function in pepper resistance to PMMoV infection remains to be investigated.

Photosynthesis is critical for growth and developmental processes in planta, and also plays an important role during plant virus infection (Cheng et al., 2008). It was suggested that mosaic or chlorosis symptoms in virus-infected leaves were caused by disruption of normal chloroplast function (Cheng et al., 2008; Song et al., 2009). Molecular studies in proteomic analyses of maize (*Zea mays*) seedlings in response to sugarcane mosaic virus (SCMV) infection revealed that most of the photosynthesis-related proteins were down-regulated with the exception of the RuBisCO large subunit and the ferredoxin-NADP reductase and its isoforms (Wu et al., 2013). Similarly, studies on *N. benthamiana* plants infected by PMMoV revealed that several proteins involved in both the photosynthetic electron-transport chain and the Benson-Calvin cycle are down-regulated during viral infection (Pineda et al., 2010). In our study, the expression levels of DEGs encoding *PetF*, *PsaA*, *PsbP*, *CP43*, *PetA* and *AtpA* in photosynthesis, *Lhcb5*, *CAB11* and *CAB13* in photosynthesis-antenna protein and *GSA* and *CPO* in porphyrin and chlorophyll metabolism were all up-regulated in resistant line 21C385 compared to susceptible line 21C241. Since chlorophyll is a key compound in green plants, this could explain, to some extent, the reason of yellowing induced due to PMMoV infection in susceptible line 21C241, but not resistant line 21C385. Regarding the pathway of the photosynthesis-antenna proteins, three genes (encoding *Lhcb5*, *CAB11* and *CAB13*) were up-regulated. Thus, the up-regulation and down-regulation of the genes mentioned above caused by PMMoV infection might be the key elements contributing to the yellowing symptoms development observed in susceptible line 21C241.

Light capture is the most important step in photosynthesis, which is mediated by light capture chlorophyll a/b binding proteins (Hey and Grimm, 2018; Wei et al., 2022). These proteins are plant-specific families, mainly locating on the chloroplast thylakoid membrane and combining with pigments to form a light-harvesting pigment-protein complex, which are involved in the collection and transmission of light energy during photosynthesis (Liu et al., 2008; Wei et al., 2016). It also plays an important role in plant’s response to biotic stresses. For instance, SA increased RuBisCo and stabilized chlorophyll a-b binding protein expression, increased plant height and root volume, delayed symptom expression, and reduced infection rate during maize dwarf mosaic virus infection (Li et al., 2020). Four genes encoding photosystem I chlorophyll a/b-binding protein 6 (*Lhca2*), chlorophyll a-b binding protein of LHCII type III (*Lhcb3*) and chlorophyll a-b binding protein CP24 (*Lhcb6*) were consistently and greatly decreased during wheat dwarf virus infection, which may lead to decreased photoelectron capture and photosynthetic ability in wheat leaves following infection (Liu et al., 2020). Interestingly, our results also showed that knockdown of *CAB13* expression inhibits PMMoV infection in pepper and *N. benthamiana*. These findings indicate that PMMoV infection may lead to decreased photoelectron capture and photosynthetic ability. Consequently, this might affect the enzymes involved in photosynthesis, alerting the structure and function of chloroplasts during PMMoV infection. Further studies are needed to elucidate the intracellular mechanism and network underlying *CAB13* expression changes in regulating PMMoV infection.

## Data Availability

The original contributions presented in the study are includedin the article/**Supplementary Material**. Further inquiries can bedirected to the corresponding authors.
