# Supplementary material for: The transcriptional analysis of pepper shed light on a proviral role of light-harvesting chlorophyll a/b binding protein 13 during infection of pepper mild mottle virus
Source: Front Plant Sci. 2025 Jan 27;16:1533151. doi: 10.3389/fpls.2025.1533151 (PMC11808148; doi:10.3389/fpls.2025.1533151)
Supplement: Supplementary file 1 [file Image1.pdf]

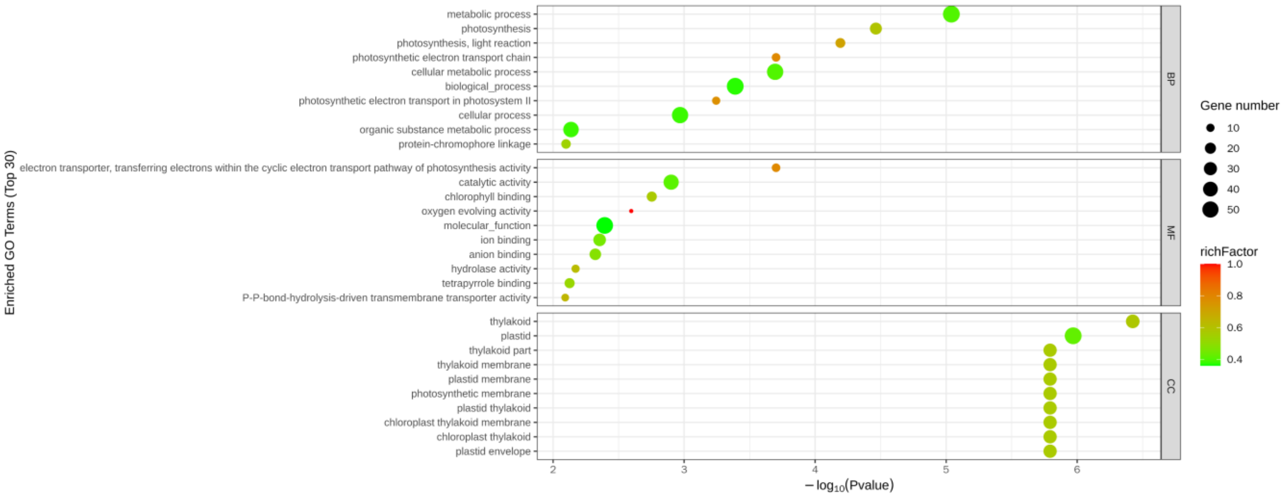

Fig. S1 Gene Ontology (GO) analyses of DEGs responsive to PMMoV infection 21C385 and 21C241 pepper plants (*P* < 0.05).

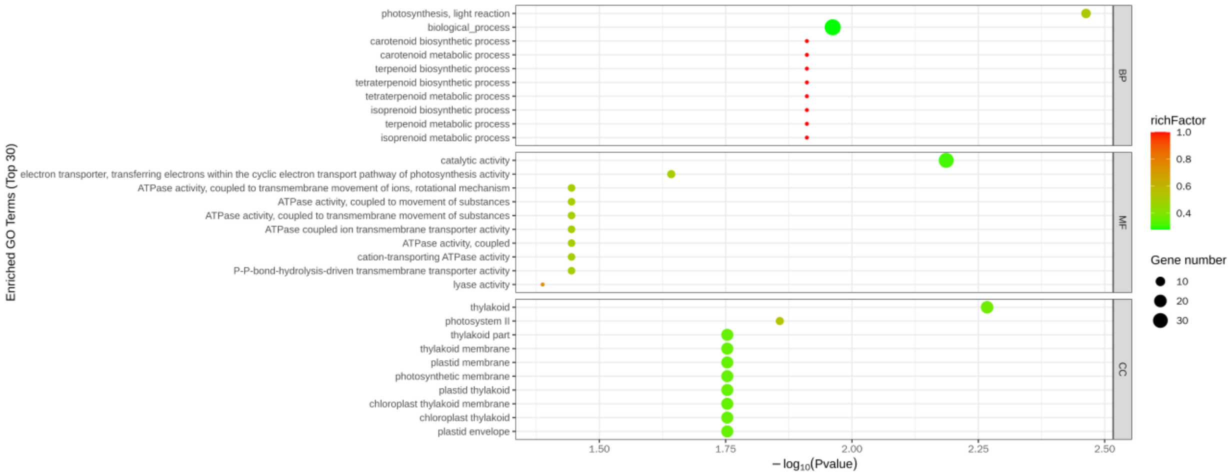

Fig. S2 Gene Ontology (GO) analyses of DEGs responsive to healthy 21C385 and 21C241 pepper plants (*P* < 0.05).

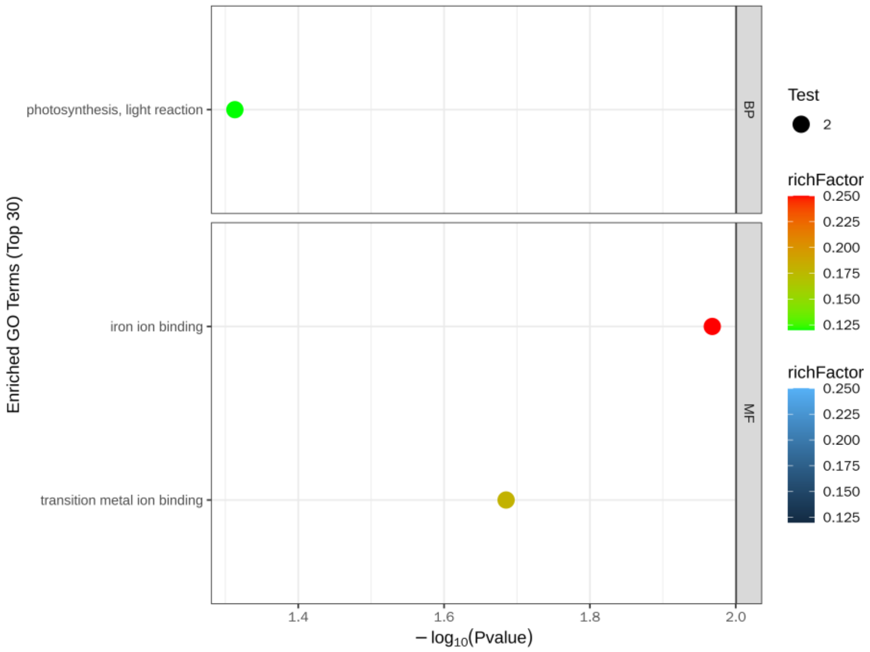

Fig. S3 Gene Ontology (GO) analyses of DEGs responsive to PMMoV infection in PMMoV-infected and healthy 21C241 pepper plants (*P* < 0.05).

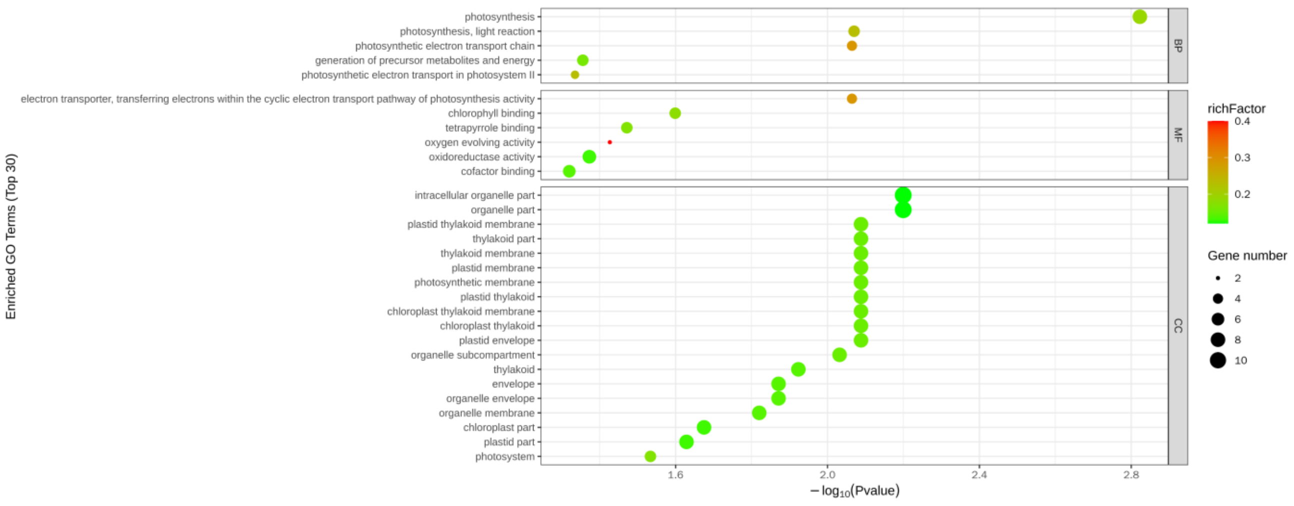

Fig. S4 Gene Ontology (GO) analyses of DEGs responsive to PMMoV infection in PMMoV-infected and healthy 21C385 pepper plants (*P* < 0.05).

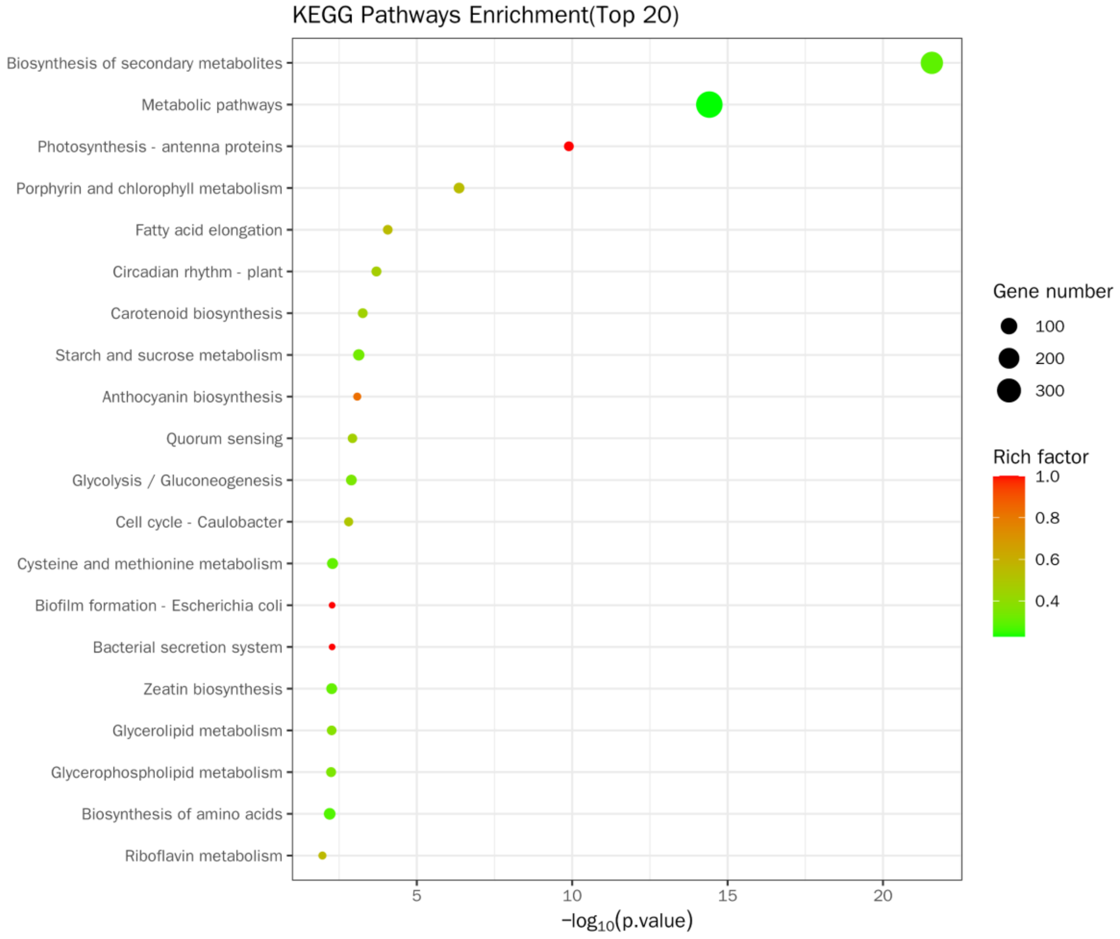

Fig. S5 Kyoto Encyclopedia of Genes and Genomes (KEGG) analyses of DEGs responsive to PMMoV infection 21C385 and 21C241 pepper plants.

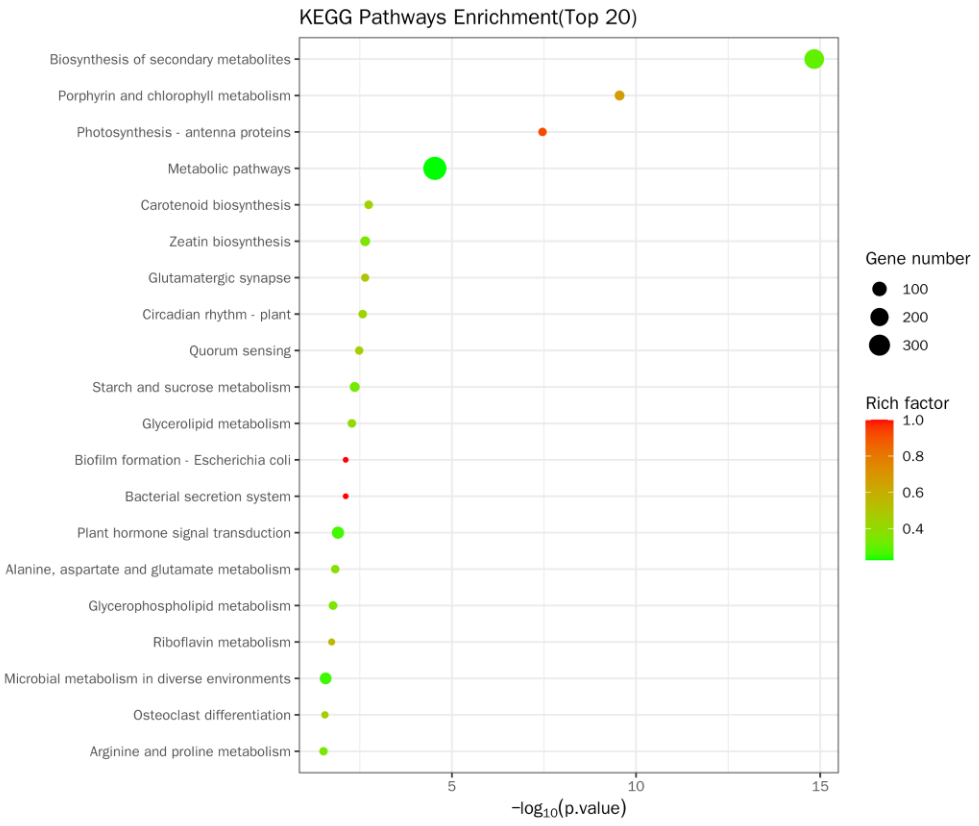

Fig. S6 Kyoto Encyclopedia of Genes and Genomes (KEGG) analyses of DEGs responsive to healthy 21C385 and 21C241 pepper plants (*P* < 0.05).

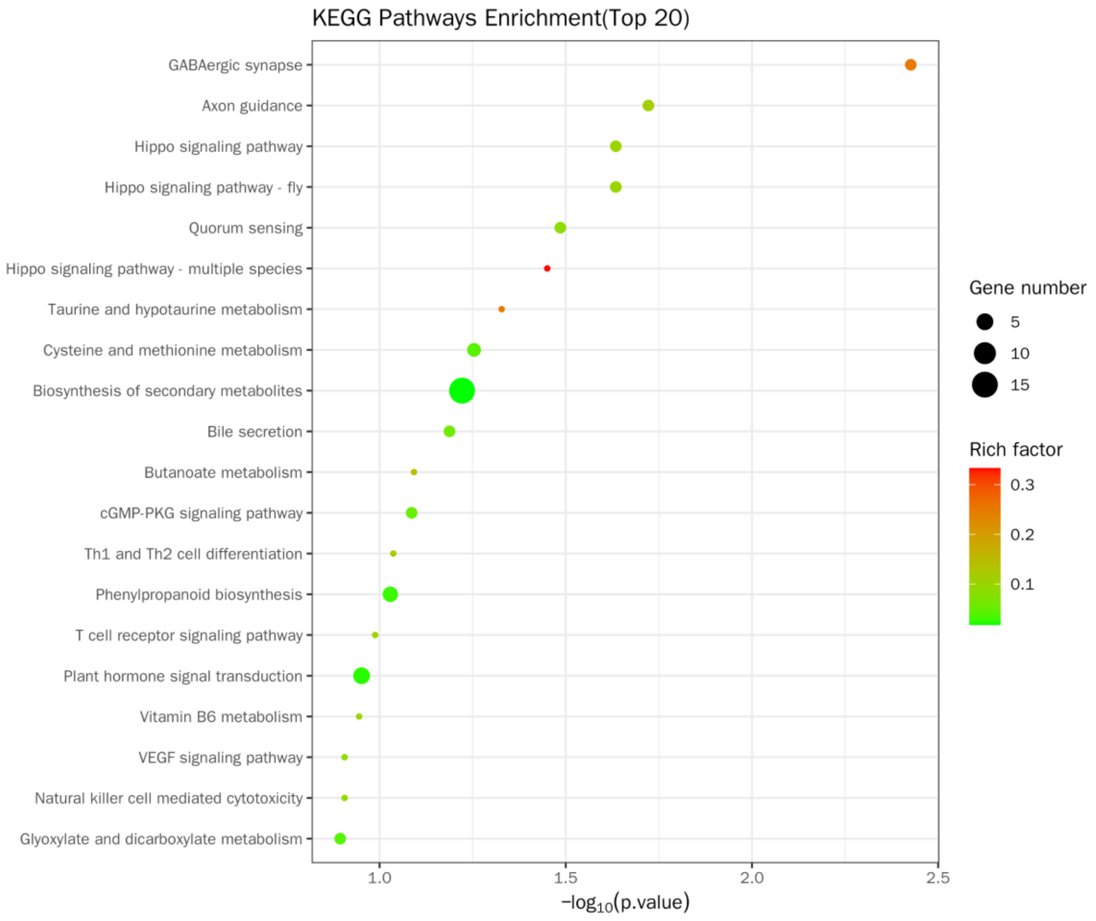

Fig. S7 Kyoto Encyclopedia of Genes and Genomes (KEGG) analyses of DEGs responsive to PMMoV infection in PMMoV-infected and healthy 21C241 pepper plants.

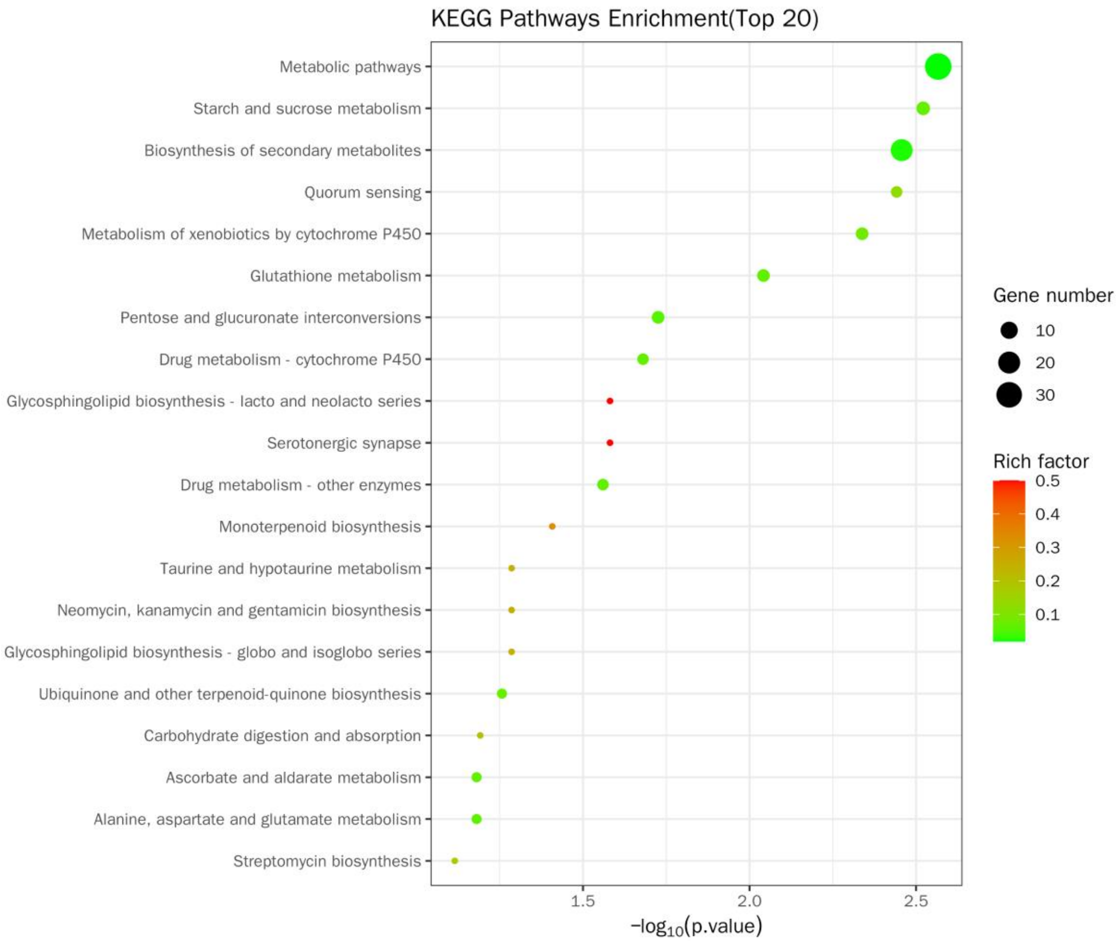

Fig. S8 Kyoto Encyclopedia of Genes and Genomes (KEGG) analyses of DEGs responsive to PMMoV infection in PMMoV-infected and healthy 21C385 pepper plants.

```
CaCAB13.seq    ATGGCATCAATGGCAGCAACAGCTAGCTCCACCACAGTTG     40
NbCAB13.seq    ATGGCATCAATGGCAGCAACAGCAAGCTCCACCACAGTTG     40
Consensus      atggcatcaatggcagcaacagc agctccaccacagttg

CaCAB13.seq    TTAGAGCAACTCCATTCTTGGGTCAAACCAAGAATACTAA     80
NbCAB13.seq    TTAGAGCAACCCCATTTTTGGGCCAAACCAAGAATGCTAA     80
Consensus      ttagagcaac ccatt ttggg caaaccaagaat ctaa

CaCAB13.seq    CCCTCTTAGAGATGTTGTTCCCATGGGCTCTGGCAAATTT    120
NbCAB13.seq    CCCTCTTAGAGATGTTGTTCCCATGGGCTCTGCCAAATTC    120
Consensus      ccctcttagagatgttgttcccatgggctctg caaatt

CaCAB13.seq    ACCATGAGTAATGATTTGTGGTATGGACCAGACCGTGTCA    160
NbCAB13.seq    ACCATGGGTAATGACTTATGGTATGGACCCGACCGTGTCA    160
Consensus      accatg gtaatga tt tggtatggacc gaccgtgtca

CaCAB13.seq    AGTACTTGGGACCCTTTTCTGCTCAAACTCCTTCATACTT    200
NbCAB13.seq    AGTACTTGGGACCCTTTTCAGCTCAAACTCCTTCGTACTT    200
Consensus      agtacttgggacccttttc gctcaaactccttc tactt

CaCAB13.seq    GACCGGAGAATTCCCTGGTGATTACGGATGGGACACTGCT    240
NbCAB13.seq    GACTGGTGAATTCCCTGGTGATTACGGATGGGACACTGCT    240
Consensus      gac gg gaattccctggtgattacggatgggacactgct

CaCAB13.seq    GGTTTATCTGCTGATCCCGAGGCCTTTGCCAAGAACAGAG    280
NbCAB13.seq    GGTTTATCCGCTGATCCTGAGGCTTTTGCCAAGAACAGAG    280
Consensus      ggtttatc gctgatcc gaggc tttgccaagaacagag

CaCAB13.seq    CTCTTGAGGTTATCCATGGGAGATGGGCAATGCTTGGTGC    320
NbCAB13.seq    CTCTTGAGGTTATCCATGGTAGATGGGCAATGCTTGGAGC    320
Consensus      ctcttgaggttatccatgg agatgggcaatgcttgg gc

CaCAB13.seq    TCTAGGTTGCATTACCCCAGAAGTTCTTGAAAAATGGGTG    360
NbCAB13.seq    ACTAGGTTGCATCACCCCAGAAGTTCTTGAAAAATGGGTG    360
Consensus       ctaggttgcat accccagaagttcttgaaaaatgggtg

CaCAB13.seq    AAGGTGGACTTCAAAGAACCAGTATGGTTCAAAGCAGGAT    400
NbCAB13.seq    AAAGTGGACTTCAAAGAACCAGTATGGTTCAAAGCAGGAG    400
Consensus      aa gtggacttcaaagaaccagtatggttcaaagcagga

CaCAB13.seq    CACAAATCTTCAGTGACGGTGGACTTGACTACTTGGGCAA    440
NbCAB13.seq    CTCAAATCTTCAGCGAAGGGGGACTTGACTACTTAGGCAA    440
Consensus      c caaatcttcag ga gg ggacttgactactt ggcaa

CaCAB13.seq    CCCCAACCTTGTCCATGCTCAGAGTATCCTAGCTGTGCTA    480
NbCAB13.seq    CCCTAACCTTGTGCATGCTCAGAGTATTCTAGCTGTGCTA    480
Consensus      ccc aaccttgt catgctcagagtat ctagctgtgcta

CaCAB13.seq    GGTTTCCAAGTGGTGCTTATGGGACTTGTTGAAGGTTTCA    520
NbCAB13.seq    GGTTTTCAAGTTGTGCTCATGGGACTTGTTGAAGGTTTTA    520
Consensus      ggttt caagt gtgct atgggacttgttgaaggttt a

CaCAB13.seq    GAATTAATGGACTTCCCGGAGTTGGAGAAGGCAACGATTT    560
NbCAB13.seq    GAATTAATGGACTTCCTGGAGTCGGAGAAGGCAACAACTT    560
Consensus      gaattaatggacttcc ggagt ggagaaggcaac a tt

CaCAB13.seq    ATACCCTGGCGGCCAATACTTTGACCCACTCGGCTTAGCT    600
NbCAB13.seq    ATACCCTGGTGGTCAATATTTTGACCCACTTGGCCTAGCT    600
Consensus      ataccctgg gg caata tttgacccact ggc tagct

CaCAB13.seq    GATGACCCTACAACATTTGCAGAACTCAAGGTGAAGGAGA    640
NbCAB13.seq    GATGACCCGACAACTTTTGCCGAGCTCAAGGTGAAGGAGA    640
Consensus      gatgaccc acaac tttgc ga ctcaaggtgaaggaga

CaCAB13.seq    TCAAGAACGGAAGATTGGCGATGTTCTCCATGTTCGGATT    680
NbCAB13.seq    TCAAGAACGGAAGATTGGCTATGTTCTCCATGTTCGGATT    680
Consensus      tcaagaacggaagattggc atgttctccatgttcggatt

CaCAB13.seq    CTTCGTTCAAGCTATTGTCACCGGCAAAGGCCCTCTTGAG    720
NbCAB13.seq    CTTTGTTCAAGCTATTGTCACCGGAAAAGGCCCTCTTGAG    720
Consensus      ctt gttcaagctattgtcaccgg aaaggccctcttgag

CaCAB13.seq    AACCTATTGGACCACCTTGACAACCCTGTTGCTAATAATG    760
NbCAB13.seq    AACCTATTGGATCACCTTGACAACCCTGTTGCTAACAATG    760
Consensus      aacctattgga caccttgacaaccctgttgctaa aatg

CaCAB13.seq    CTTGGGTTTATGCCACTAAATTTGTTCCTGGATCTTA       797
NbCAB13.seq    CTTGGGTTTATGCTACAAAGTTTGTTCCTGGAGCTTA       797
Consensus      cttgggtttatgc ac aa tttgttcctgga ctta
```

Fig. S9 Nucleotide sequence alignment of *CAB13* from *Capsicum annuum* and *N. benthamiana*. *NbCAB13* shares 92.73% nucleotide identity with *CaCAB13*.

```
CaCAB13.seq     MASMAATASSTTVVRATPFLGQTKNTNPLRDVVPMGSGKF     40
NbCAB13.seq     MASMAATASSTTVVRATPFLGQTKNANPLRDVVPMGSAKF     40
Consensus       masmaatassttvvratpflgqtkn nplrdvvpmgs kf

CaCAB13.seq     TMSNDLWYGPDRVKYLGPFSAQTPSYLTGEFPGDYGWDTA     80
NbCAB13.seq     TMGNDLWYGPDRVKYLGPFSAQTPSYLTGEFPGDYGWDTA     80
Consensus       tm ndlwygpdrvkylgpfsaqtpsyltgefpgdygwdta

CaCAB13.seq     GLSADPEAFAKNRALEVIHGRWAMLGALGCITPEVLEKWV    120
NbCAB13.seq     GLSADPEAFAKNRALEVIHGRWAMLGALGCITPEVLEKWV    120
Consensus       glsadpeafaknralevihgrwamlgalgcitpevlekwv

CaCAB13.seq     KVDFKEPVWFKAGSQIFSDGGLDYLGNPNLVHAQSILAVL    160
NbCAB13.seq     KVDFKEPVWFKAGAQIFSEGGLDYLGNPNLVHAQSILAVL    160
Consensus       kvdfkepvwfkag qifs ggldylgnpnlvhaqsilavl

CaCAB13.seq     GFQVVLMGLVEGFRINGLPGVGEGNDLYPGGQYFDPLGLA    200
NbCAB13.seq     GFQVVLMGLVEGFRINGLPGVGEGNNLYPGGQYFDPLGLA    200
Consensus       gfqvvlmglvegfringlpgvgegn lypggqyfdplgla

CaCAB13.seq     DDPTTFAELKVKEIKNGRLAMFSMFGFFVQAIVTGKGPLE    240
NbCAB13.seq     DDPTTFAELKVKEIKNGRLAMFSMFGFFVQAIVTGKGPLE    240
Consensus       ddpttfaelkvkeikngrlamfsmfgffvqaivtgkgple

CaCAB13.seq     NLLDHLDNPVANNAWVYATKFVPG                    264
NbCAB13.seq     NLLDHLDNPVANNAWVYATKFVPG                    264
Consensus       nlldhldnpvannawvyatkfvpg
```

Fig. S10 Amino acid sequence alignment of CAB13 from *Capsicum annuum* and *N. benthamiana*. NbCAB13 shares 97.36% amino acid identity with CaCAB13.
